# Socioeconomic Status Modifies the Seasonal Effect on Blood Pressure: Findings From a National Panel Study

**DOI:** 10.1097/MD.0000000000001389

**Published:** 2015-09-04

**Authors:** Annibale Cois, Rodney Ehrlich

**Affiliations:** From the Division of Epidemiology and Biostatistics (AC); and Centre for Environmental and Occupational Health Research, School of Public Health and Family Medicine, University of Cape Town, Cape Town, South Africa (RE).

## Abstract

Seasonal variations in blood pressure have been consistently reported. However, uncertainty remains about the size of the seasonal effect in different regions, and about factors that explain the differences observed across and within populations. Using data from a national panel study, we investigated seasonal variations in blood pressure in the South African adult population, and whether these variations differed across socioeconomic strata.

We estimated age-specific seasonal effects on blood pressure using a multilevel structural equation model, with repeated measurements nested within subjects. Effect modification by socioeconomic status was assessed by repeating the analyses in the subpopulations defined by levels of education, household income per capita, and type of housing.

In men and women, season had a statistically significant effect on blood pressure, with higher levels in winter and lower levels in summer. For systolic blood pressure, the magnitude of the seasonal effect was 4.25/4.21 mmHg (women/men) and was higher in the older age groups. For diastolic blood pressure, the effect size was 4.00/4.01 mmHg, with no evident age trend. Seasonal effects were higher among subjects in the lowest socioeconomic classes than in the highest, with differences between 2.4 and 7.7 mmHg, depending on gender, whether systolic or diastolic blood pressure, and socioeconomic status indicator.

In the South African adult population, blood pressure shows seasonal variation modified by age and socioeconomic status. These variations have epidemiological, clinical, and public health implications, including the prospect of population level intervention to reduce elevated risk of cold weather cardiovascular morbidity.

## Abstract 2

Supplemental Digital Content is available in the text

## INTRODUCTION

A large number of studies have consistently observed winter peaks and summer troughs in blood pressure values, in clinical,^1–4^ general,^5–7^ and special populations such as children^8^ and pregnant women.^9,10^

The study of seasonal variation of blood pressure in various settings is of substantial clinical and public health interest, not least because such variation mirrors seasonal variations in cardiovascular morbidity and mortality. The evidence of this relationship is especially strong for some pathologies directly associated with hypertension, such as hemorrhagic stroke, whose incidence is higher in winter than in summer.^6,11^

The size or amplitude of the seasonal effect (measured as the difference between the winter peak and summer trough of the population averaged annual cycle) varies across populations and studies. Average effects across 24 adult population surveys in 15 countries have been calculated in a recent meta-analysis by Marti-Soler et al.^12^ The results confirm the existence of a clear seasonal pattern in blood pressure, with higher values consistently recorded in winter and lower values in summer. In the Northern Hemisphere, the magnitude of the pooled effect was 2.93 and 1.32 mmHg, respectively, for systolic (SBP) and diastolic blood pressure (DBP). In the Southern Hemisphere, the values were 3.44 and 0.86 mmHg. A previous joint analysis of the SBP data collected in 25 populations during the WHO's MONItoring of trends and determinants in CArdiovascular disease (MONICA) Project found a slightly lower pooled effect of 2.01 mmHg (95% Bayesian posterior interval: 1.05–3.08 mmHg).^13^ Studies that reported separate estimates for age categories have also shown consistently that the seasonal effect tends to increase with age.^1,2,7,14,15^ Gender differences have also been repeatedly observed, with varying patterns by population, clinical status, and age, but generally showing a slightly higher seasonal effect in men.^1,6,7^

The causal mechanisms underlying these variations remain unclear, but substantial evidence points to the seasonal variation of outdoor temperature as the main driver of the seasonal variability of blood pressure, possibly accompanied by an independent effect of the varying number of daylight hours.^16–18^

However, while the overall evidence of seasonal variations in blood pressure and the major causative role of temperature is strong and widely acknowledged, substantial uncertainty remains about the size of the effect in different parts of the world, especially in low- and middle-income countries. In particular, data from sub-Saharan Africa are lacking. Neither of the cited reviews includes studies from this large region with a population of 960 million and covering 47 countries. To our knowledge, only a few small-scale cross-sectional studies of this region have addressed the subject of seasonal variation in blood pressure,^19–21^ while others have addressed it only indirectly, through its effects on hypertension-related morbidity and mortality.^22–25^

Also poorly understood are the factors that, beyond climatic differences, explain the large differences observed in seasonal effect across and within populations. Among other biological, environmental, and behavioral factors, various authors have suggested that individual socioeconomic status may play a sizable role as an effect modifier of the relationship between season and blood pressure. In particular, it has been suggested that individuals with low socioeconomic status may have both a restricted access to adequate means of protection from low temperatures (eg, sufficient heating at home and adequate clothing) and working conditions which require more time outdoors than subjects with higher socioeconomic status. This would translate into a higher exposure to winter–summer temperature differences and, consequently, higher variations in blood pressure.^7,26^ Despite the plausibility of this hypothesis and some evidence that the availability of indoor temperature control attenuates the difference between winter and summer blood pressure,^14,27^ to our knowledge a direct estimation of the modifying effect of individual socioeconomic status on the magnitude of the seasonal effect on blood pressure is lacking.

This study aimed to narrow these knowledge gaps by estimating the magnitude of the seasonal variation in blood pressure in the adult population of South Africa – a middle-income country in sub-Saharan Africa characterized by high level of socioeconomic inequality – and by testing the hypothesis of an inverse relationship between seasonal effect and socioeconomic status as measured by education and household income.

The study used data from the first 3 waves of the National Income Dynamics Study (NIDS), a panel survey of individuals randomly selected in 2008 and successively recontacted in 2010 and 2012.

## METHODS

### Participants

The NIDS is a nationally representative panel survey of 28,255 South Africa's residents.^28^ The first wave of the survey was conducted in 2008, and the target population was private households and residents in workers’ hostels, convents, and monasteries. A 2-stage cluster sample design was used to randomly select about 7300 households across 400 primary sampling units (areas), stratified by district council (a second level administrative division of South Africa's territory into 53 areas). Trained fieldworkers were instructed to interview and collect anthropometric data on all available subjects belonging to the selected households. The same individuals were recontacted in the 2 subsequent waves, in 2010 and 2012, and administered the same questionnaire. The household level response rate for the first wave was 69%, and the individual response rate within households was 93%. The individual attrition rate was 19% between wave 1 and wave 2, and 16% between wave 2 and wave 3.

The NIDS study has been granted ethical approval by the Commerce Faculty Ethics Committee at the University of Cape Town, and its datasets are publicly available for research purposes.^29^ All participants received an information sheet with their blood pressure readings, and those with elevated readings were advised of the risks and of the need to seek medical attention.

Out of the 18,526 participating individuals who were 15-years old or over at the time of the first interview, this study considers the 11,440 individuals successfully reinterviewed both in the second and the third wave. Sampling weights were adjusted to take into account unequal response rates across population strata.^30^ Wave 1 dataset version 5.2, wave 2 version 2.2, and wave 3 version 1.2 were used in the analyses.

### Measures

#### Sociodemographic Variables

Age in years was categorized into 6 groups. Education was measured in years of completed schooling and categorized as primary, secondary, tertiary, and none. Place of residence was categorized as urban/rural according to Statistics South Africa's Census 2001.^31^ Urban areas were further classified as formal or informal. (According to Statistics South Africa, an informal settlement is “An unplanned settlement on land which has not been surveyed or proclaimed as residential, consisting mainly of informal dwellings (shacks).") Household monthly income per capita was calculated as the summation of a wide array of sources, as detailed by Argent.^32^

#### Blood Pressure

Supine blood pressure was measured twice by trained fieldworkers in the left arm after a 5 minute rest period, using an automated blood pressure monitor (Omron M7 BP, multisize cuff, factory calibrated). Measurements were retained if SBP was between 80 and 240 mmHg, DBP ≥ 30 mmHg, and their difference ≥15 mmHg.

#### Time

Year, date, and month of blood pressure measurement were recorded and used to create a new variable, day of measurement, representing the number of days since the first of January regardless of the year.

### Other Measurements

Duplicate measures of weight and height were recorded, with a third measure taken if their difference was greater than 0.5 kg or 0.5 cm, respectively. Excluding measures with implausible values (height <60 or >230 cm, weight <30 or >250 kg), the average of the available readings was used to calculate body mass index (BMI) in kg/m^2^. BMI was then categorized in 4 classes according to the World Health Organization's cut-off points.

Current smoking, use of antihypertensives, and past diagnosis of hypertension by a health professional were self-reported by subjects in response to direct questions.

Controlled hypertension was defined as having a past diagnosis of hypertension but readings of blood pressure within the normal range (SBP < 140 mmHg and DBP < 90 mmHg).

### Statistical Analyses

Sample characteristics were described as the median and interquartile range for continuous variables and frequency for categorical measures.

We estimated simultaneously the association between SBP and DBP with day of measurement using a multilevel linear structural equation model, with measurements at each wave nested within subjects. Considering that the relationships between many of the variables involved in the model have been shown to differ among males and females, models were fit separately by gender.

To minimize bias due to measurement error, SBP, and DBP were introduced in the models as latent variables, with the observed multiple readings as indicators.^33,34^

Seasonal effect for individual i at wave j was modeled using a trigonometric spline^35^ in the form: 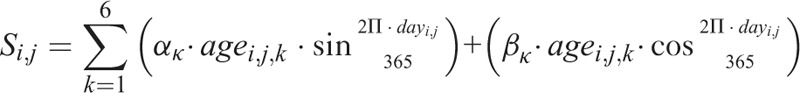


where α_k_ and β_k_ are coefficients estimated in the model, age_i,j,k_ are the dummy variables used to code the 6-age classes, and day_i,j_ represents the day of measurement for individual i at wave j, relative to the first of January.

Trigonometric splines are frequently used in epidemiological studies to model seasonal patterns, and they have been previously applied in the study of seasonal variations in blood pressure.^5,12,13^ Our specific implementation, including interaction terms for each age category, allowed for the estimation of the magnitude of the seasonal effect independently for each age category. Overall effects were obtained as weighted averages of the age-specific estimates, with weights reproducing the age structure of the South African population in 2011.

Categorical age, urban or rural place of residence, categorical BMI, and smoking status were introduced in the model as occasion-dependent covariates.

Adjustment for the effect of antihypertensive treatment was done adding a constant (10 mmHg for SBP and 5 mmHg for DBP) to the observed readings of treated individuals.^36^ The sensitivity of the estimates to the exact values of the constants was assessed by replicating the model estimation with different values for the constants. The estimation was also replicated excluding treated individuals from the dataset.

Random intercepts were used to take into account differences in average blood pressure values across individuals, while the coefficients α_k_ and β_k_, which defined the seasonal effects and all other coefficients, were considered as fixed.

Missing data in the outcome variables were addressed by estimating model coefficients by full-information maximum likelihood, which provides asymptotically unbiased estimates under the hypothesis that data are missed at random, conditional on the observed covariates.

Effect modification by socioeconomic status was assessed by repeating the analyses in the subpopulations defined by level of education, tertile of household monthly income per capita, and formal versus informal housing type (restricted to urban settlements). The existence of a statistically significant monotonic trend in the seasonal effect across increasing levels of education and income was tested by simulation, adapting the procedure proposed by Soderberg and Hennet.^37^

For illustrative purposes, a simulated effect attributable to seasonal variations in blood pressure was modeled by calculating the absolute difference of 10-year risk of developing any major atherosclerotic cardiovascular disease when the values of blood pressure vary according to the estimated seasonal effect. The simulations were conducted for different subpopulations using the Framingham study general cardiovascular risk equations.^38^

Significance level for hypothesis testing was set at α = 0.05. Statistical calculations were carried out using R Statistical Environment v. 3.0.2 (R core Team, Vienna) and Mplus v. 7.3 (Muthén &Muthén, LA).

Further details on modeling assumptions are provided as supplementary material, http://links.lww.com/MD/A387.

## RESULTS

Unweighted sample characteristics at wave 1 are described in Table 1.

**TABLE 1:**
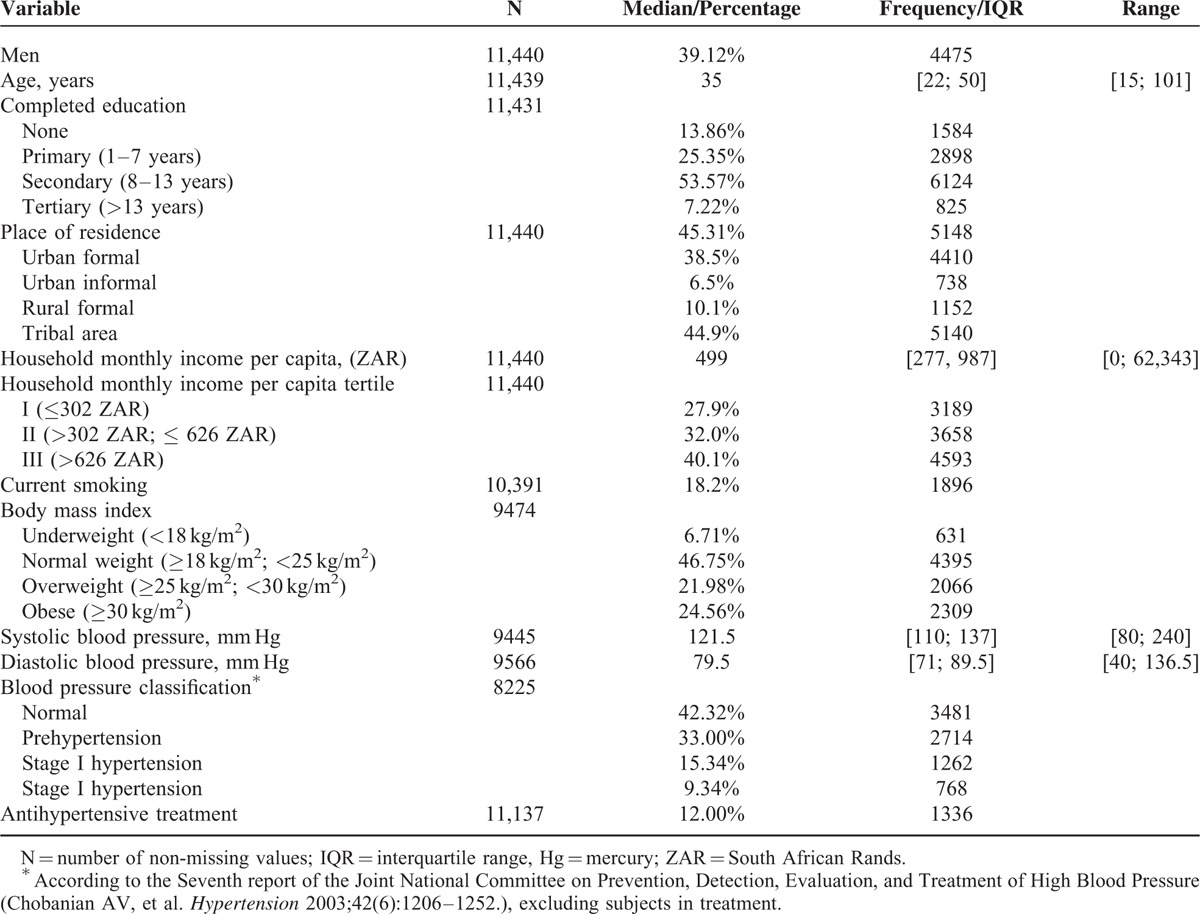
Sample Descriptive Statistics at Baseline

Population strata of higher socioeconomic status were underrepresented relative to the South African population, owing to their low response rate in the first wave and their higher attrition than the other population groups.

Current use of antihypertensives was reported by 6% of men and 16% of women. Using SBP ≥ 140 mmHg and/or DBP ≥ 90 mmHg as cut-offs, 20.0% of untreated male participants and 21.7% of untreated female participants would be classified as hypertensive.

Additional Table 1 and 2, http://links.lww.com/MD/A387 in the supplementary material, http://links.lww.com/MD/A387 provide sample descriptive statistics at wave 2 and 3 and distribution of subjects by month of data collection.

### Seasonal Effects

In both genders, season had a statistically significant effect on SBP and DBP.

The magnitude of the overall seasonal effect on SBP was 4.25 mmHg (95% CI: 3.18–5.31 mmHg) in women and 4.21 mmHg (95% CI: 2.98–5.44 mmHg) in men. The effect of season on DBP was slightly lower in both genders: 4.00 mmHg (95% CI: 3.21–4.78 mmHg) in women versus 4.01 mmHg (95% CI: 3.17–4.96 mmHg) among men.

**FIGURE 1:**
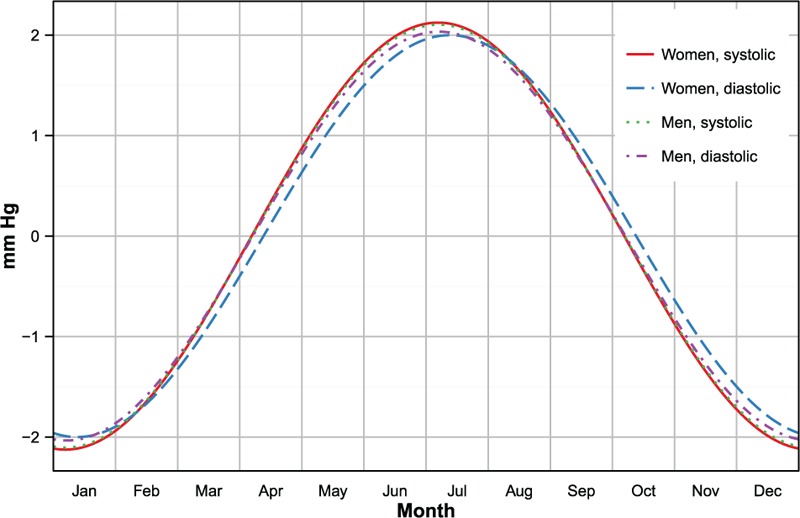
Estimated seasonal variation of blood pressure in the South African population, by gender. Values represents variations over the annual mean. Estimates are adjusted for age, urban or rural place of residence, body mass index, and smoking status.

As shown in Figure 1, the position of the overall seasonal peak (and, consequently, the position of the trough, constrained to be 6 months apart by the analytical form of the trigonometric spline) showed little variation between systolic and diastolic values and across genders. All peaks occurred in a 6 days range in the Southern Hemisphere winter, during the second week of July, in most parts of South Africa the coldest period in the year. (South Africa is situated between 22 and 35°S, in the Southern Hemisphere's subtropical zone. Temperature excursions between summer and winter are moderated by the presence of the ocean on three sides of the country and the altitude of the interior plateau. Maximum temperatures in summer (mid-October to mid-February) are usually below 30 °C, and minimum temperatures in winter (May to July) above 5°–6°.^49^) The differences in the position of the peak were not statistically significant.

**FIGURE 2:**
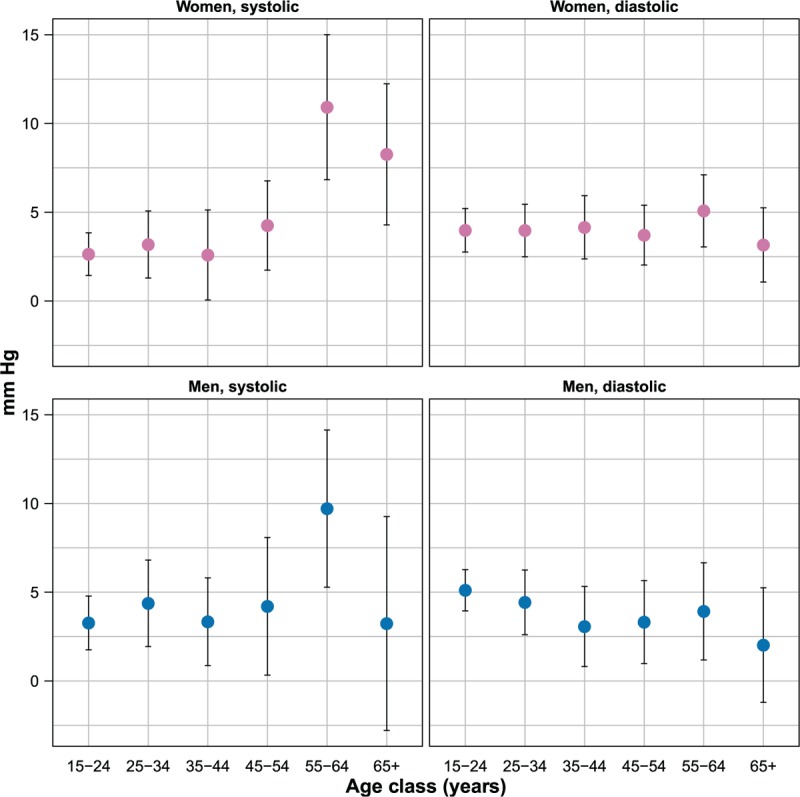
Age-specific magnitude of seasonal effect on blood pressure, by gender (estimates and 95% confidence intervals, mmHg). Estimates are adjusted for age, urban or rural place of residence, body mass index, and smoking status.

Age-specific magnitudes of seasonal effects are depicted in Figure 2. In both genders, seasonal effects on SBP increased with age, reached a maximum in the 55 to 64 years age category, and then fell among the oldest participants (65 years and above). Age-related differences in seasonal effect on DBP were much smaller, and none of them reached statistical significance.

### Seasonal Effects and Socioeconomic Status

**FIGURE 3:**
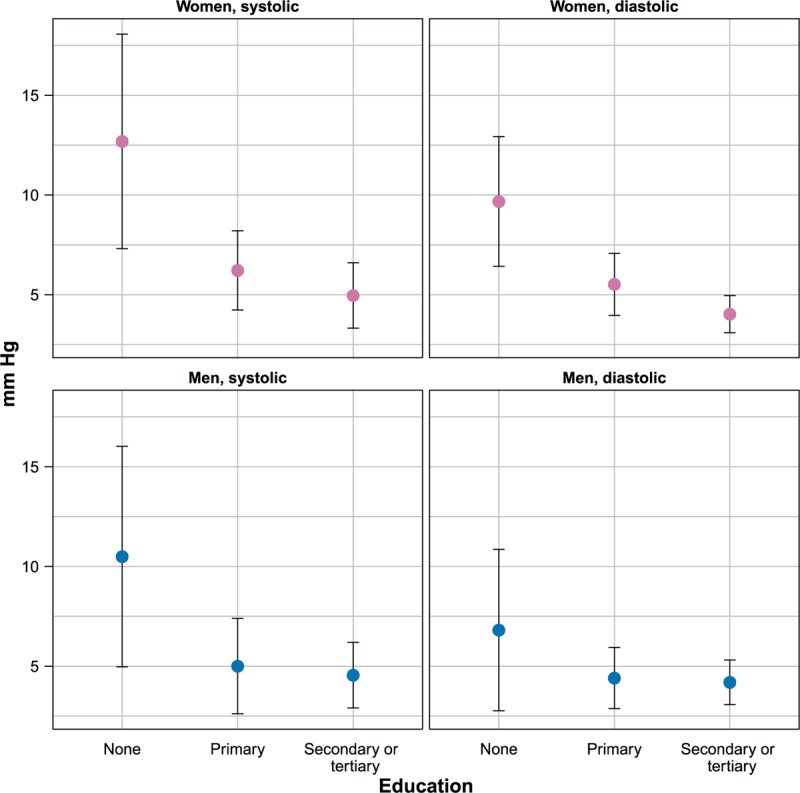
Magnitude of seasonal effect on blood pressure by education and gender (estimates and 95% confidence intervals). Estimates are adjusted for age, urban or rural place of residence, body mass index, and smoking status.

**FIGURE 4:**
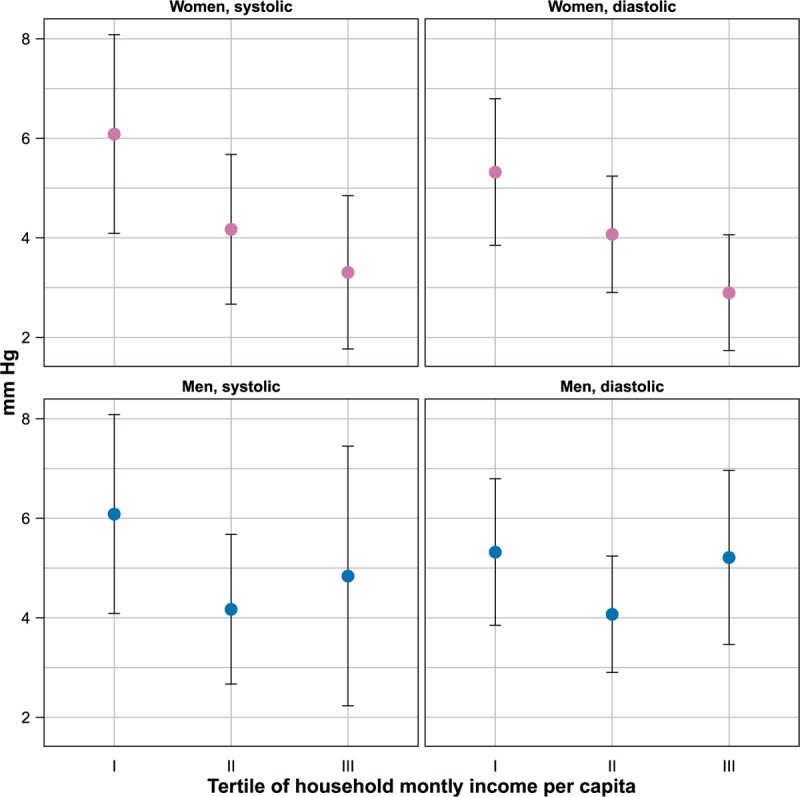
Magnitude of seasonal effect on blood pressure by household income per capita and gender (estimates and 95% confidence intervals). Estimates are adjusted for age, urban or rural place of residence, body mass index, and smoking status.

The magnitude of the age-standardized seasonal effect by socioeconomic status in selected subpopulations is depicted in Figures 3 and 4.

Among women, increasing education and household monthly income per capita were associated with a decreasing seasonal effect on both SBP and DBP. The average seasonal effect on SBP decreased by 7.7 mmHg (from 12.7 to 5.0 mmHg) going from no education to secondary education or above, while the effect on DBP decreased by 5.7 mmHg (from 9.7 to 4.0 mmHg). Differences in seasonal effects between the first and last income tertile were smaller than those for education, such as 2.8 (from 6.1 to 3.3 mmHg) in SBP and 2.4 mmHg (from 5.3 to 2.9 mmHg) in DBP.

Among men, the overall pattern of association between socioeconomic status indicators and seasonal effects was similar to that among women. Increasing education was associated with decreasing seasonal effect, with a reduction by 5.9 (from 10.5 to 4.6 mmHg) and 2.6 mmHg (from 6.8 to 4.2 mmHg) moving from the lowest to the highest education class, respectively, for SBP and DBP. The lowest income tertile was also associated with a higher seasonal effect than higher income tertiles, but the relationship was not monotonic.

Table 2 shows the estimated values for the Kendall correlation coefficients (and associated 95% confidence intervals) between seasonal effect and socioeconomic status indicators.

**TABLE 2:**
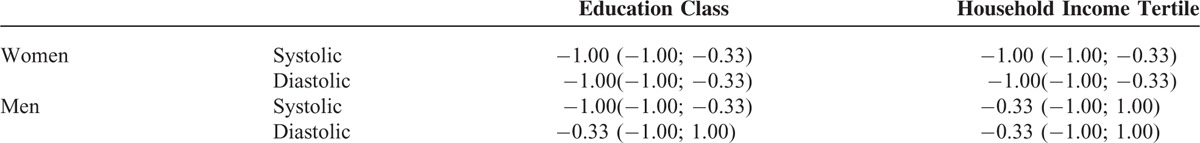
Kendall Tau Correlation Coefficients Between Seasonal Effects and Socioeconomic Status Indicators (Estimates and 95% Confidence Intervals)

Among women, all estimates for the Kendall correlation coefficients were negative and none of their 95% confidence intervals included 0, thus supporting the existence of a statistically significant monotonic decreasing trend between socioeconomic status and seasonal effects on blood pressure. Among men, the test for trend showed a statistically significant result only for the relationship between education and SBP.

Seasonal effects were higher among urban dwellers living in informal than formal settlements, both for SBP and DBP, and for men and women (see additional Table 4, http://links.lww.com/MD/A387 in the supplementary material, http://links.lww.com/MD/A387). None of the differences reached statistical significance.

### Projected Impact on Cardiovascular Risk

**FIGURE 5:**
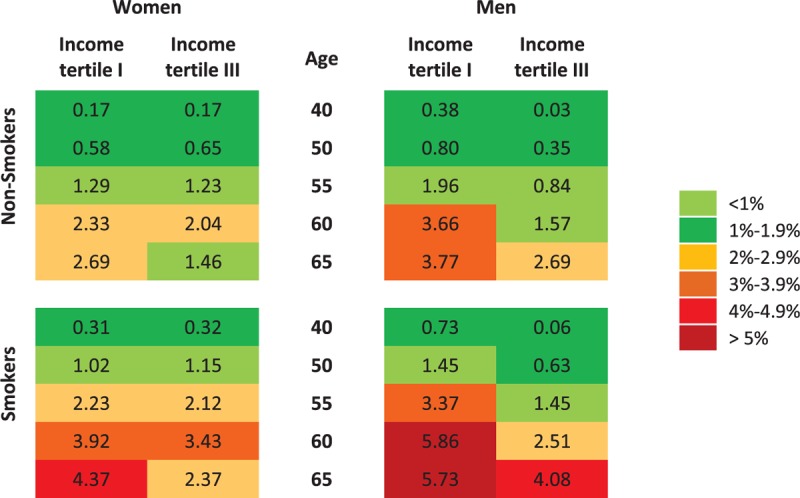
Simulated effect of seasonal variation in blood pressure on 10-year cumulative risk of developing any major atherosclerotic cardiovascular event. The chart shows the absolute difference on 10-year risk of developing any major atherosclerotic cardiovascular event when the values of blood pressure vary according to the estimated seasonal effect, by gender, age, smoking status, and income tertile. Absolute risks are calculated as in D’Agostino et al.^38^ Body mass index is kept constant at the population average, by gender and age category. Other risk factors not explicitly shown in the chart (diabetes and antihypertensive treatment) are considered to be absent.

The projected absolute difference in 10-year cumulative risk percent of developing any major atherosclerotic cardiovascular disease when the SBP varies according to the estimated seasonal effect is summarized in Figure 5, for different subpopulations. The projected excess risk between winter and summer increased with age and was higher among males, smokers, and subjects with low socioeconomic status (as indicated by low household income). Overall, the excess risk was negligible in the younger age groups, but became evident among elderly subjects, especially those with low socioeconomic status.

### Model Fit and Sensitivity Analysis

All models used for the analyses had an excellent fit the data. Fit indices for the models estimated in the different subpopulations are available as supplemental material, http://links.lww.com/MD/A387 (Additional Table 3, http://links.lww.com/MD/A387).

On repeating the analyses using different combinations of constants to adjust for antihypertensive treatment, none of the population estimates of seasonal effects changed more than 4.5%, thus supporting a relative insensitivity to the exact value of the constants. Constants were allowed to vary in the range 5 to 15 mmHg for diastolic and 10 to 20 mmHg for SBP. These ranges approximately correspond to the 95% confidence intervals of the average effects of antihypertensive treatment estimated by Wu et al^39^ in their meta-analysis of 165 clinical trials. As expected, omitting the correction produced a decrease of the estimated seasonal effect,^36^ by 0.12/0.19 mmHg (SBP/DBP) among females, and by 0.11/<0.01 mmHg among males.

Excluding subjects on hypertensive treatment produced modest changes in the values of the estimates and did not affect the conclusions of the study (Additional Table 5, http://links.lww.com/MD/A387).

## DISCUSSION

In line with the literature referring to other geographical areas, the results of this study showed a clear seasonal pattern in the values of blood pressure in the South African adult population, with higher values recorded in winter months and lower values in summer. The magnitude of the seasonal effect on both SBP and DBP was almost identical across genders. In agreement with most of the studies, the seasonal effect was more pronounced for SBP.

The commonly observed positive relationship between magnitude of the seasonal effect and age has been confirmed in our study, as well as the finding of a greater age effect on SBP than on DBP.^6,15^ In our population, the positive trend seems to be reversed in the eldest age category (>64 years). This result replicates the findings of a large cross-sectional study in China, which found that the average difference between winter and summer blood pressure increased with age up to about 70 years, and then showed a relative decrease.^14^ In another study, a more detailed analysis of the seasonal effect among older individuals (hypertensive subjects 69–91-years old, divided into 5-year age groups), found a peak in the magnitude among the 70 to 75-years old, and a progressive downward trend in the subsequent age classes, suggesting that the relative reduction of the seasonal effect continues in older ages.^3^

The causal mechanism underlying this complex relationship between age and seasonal effect on blood pressure is unclear, and it is likely to reflect a combination of factors both biological and behavioral. Previous studies have shown a decrease of autonomic response to cold,^6^ a lower ability to control deep body temperature,^40^ and a reduction of the baroreflex sensitivity^41^ with increasing age. These phenomena point to a reduced ability of older subjects to compensate for the increase in blood pressure caused by cold-induced peripheral vasoconstriction (an effect which itself does not seem to be impaired by age).^42^ This physiological “mismatch” may thus produce the positive relationship between age and seasonal effect. Increased arterial wall rigidity – which has been shown to be correlated with winter–summer differences in SBP and is strongly associated with age – could also be an important causal mediator of this relationship.^43^ A tentative explanation of the relative decrease of winter–summer differences in the oldest age groups could be related to lower exposure to outdoor temperature by the oldest subjects because of reduced working activity and deteriorating health.

In our study, the magnitude of the seasonal effect in the population as a whole was only slightly lower for diastolic than for SBP. This result is in contrast with the findings of the systematic review by Marti-Soler and other large studies, which generally indicated an average seasonal effects on DBP substantially lower than the corresponding effect on SBP.^6,7,12^ This discrepancy is not unexpected, given that the populations considered in those studies were, on average, much older that the population analyzed here. As previously observed, seasonal effects on SBP increase rapidly with age, while the same trend is less evident for DBP. Therefore, in older populations the differences between diastolic and systolic effects tend to be larger than those observed in younger samples. To test the validity of this hypothesis, we recalculated seasonal effects modifying the age structure to approximately match the population studied by Su et al.^7^ (The study of Su et al used different cut-offs to define age classes. To recreate a distribution compatible with the age categories used in our study, we hypothesized that the ages of the individual were distributed uniformly within each of the Su's classes.) As expected, the ratio between seasonal effects on SBP and DBP increased from 1.1:1 to 1.6:1, closer to the 2.5:1 ratio in their study and the 2:1 in the review by Marti-Soler et al.

Previous studies have observed that the negative relationship between outdoor temperature and blood pressure is mitigated in populations with good access to central heating at work and at home, thus suggesting socioeconomic status as a plausible effect modifier of the relationship between season and blood pressure.^14,27^ Our results support this hypothesis, providing evidence of an inverse relationship between magnitude of seasonal effect and education and household income, both commonly used indicators of socioeconomic status. The finding that seasonal effect is lower among urban dwellers living in formal settlements than those in informal settlements, lends some support to the hypothesis that housing conditions contribute to this effect.

The effect modification appears to be stronger in women than in men, and the reasons of this discrepancy warrant further investigation.

The findings of this study have implications for epidemiological, clinical, and public health practice.

For epidemiological investigation, the magnitude of the seasonal effect strongly suggests that – in South Africa as in the rest of the world – studies involving the estimation of blood pressure and prevalence of hypertension should routinely take into account the period of data collection, especially when comparison with other studies is involved. Ignoring this phenomenon could bias the results, particularly if the period of data collection is restricted to a single season, as was the case of the third wave of the NIDS, where almost 50% of subjects were interviewed in winter between June and August, and none between January and March. In this case, for example, adjustment for seasonality produced a reduction of the prevalence of hypertension measured in this way among South African adults by 2 percentage points (30.9%–28.9%) in males and 1.3 percentage points (36%–34.7%) in females. (Adjustment for seasonality was done by randomly redistributing the period of data collection across the year, adjusting the individual values of blood pressure according to the average seasonal effect and recalculating the proportion of hypertensive subjects with the modified values of systolic and diastolic blood pressure.) In absolute numbers, the restriction of the data collection to the winter season produced an overestimation of the number of hypertensive adults by almost 600,000, relative to the projected result were data collection spread across the seasons. (The estimation is based on the South African adult population as per Census 2011.^50^)

Taking the seasonal variation of blood pressure into account during routine clinical practice may improve the management of hypertensive (and prehypertensive) patients. The prevalence of controlled hypertension has been previously found to have a clear seasonal pattern, deteriorating in winter, which not surprisingly mirrors the fluctuations in blood pressure.^7^ Overall, our data support the existence of this seasonal pattern, that is, poorer control in winter than summer (see Additional Figure 1, http://links.lww.com/MD/A387 in supplemental material, http://links.lww.com/MD/A387), suggesting that average current clinical practices are not sufficiently responsive to the seasonal modification of the patient's blood pressure levels. The last edition of the South African hypertension guidelines acknowledges the effects of temperature on blood pressure measurement, but makes no provision for the modification of diagnostic criteria and treatment in relation to season.^44^ The large seasonal variations observed especially in the older age groups suggest the need for seasonal modification of diagnostic and therapeutic clinical practice – not least because of the cited evidence of a direct correlation between winter increase of blood pressure and cardiovascular morbidity.

Finally, the seasonal variations in blood pressure translated into nonnegligible differences by season in the projected 10-year risk of cardiovascular disease, especially in elderly subjects with low socioeconomic status where the excess risk (cumulative incidence) in winter compared with summer ranges from 2.6% (women, nonsmokers) to 5.7% (men, smokers).

Although the simulations were performed mainly for illustrative purposes, and the winter increase in blood pressure may not have the same predictive meaning as long-term or chronic elevation, at population level the projection is consistent with the substantial evidence of higher cardiovascular mortality in colder months.^45^

In our study, the pattern of association between estimated cardiovascular risk, age, and socioeconomic status mimics previously observed pattern in mortality for cardiovascular diseases, where the winter–summer differences increased with age and decreasing education.^46^ These results further suggest that seasonal variations in blood pressure could contribute to the explanation of seasonal variations in cardiovascular mortality.

The greater seasonal effect observed among subjects with low socioeconomic status and living in informal settlements – which translates directly in increased winter–summer excess in cardiovascular risk – has public health implications. It suggests that improving people's housing conditions and ability to protect themselves from low winter temperatures may have a positive effect on hypertension prevalence and cardiovascular morbidity and mortality. This is especially true for older subjects, where seasonal effects and excess risk appear to be amplified.

## STRENGTHS

Strengths of the present study include the large sample with a broad age distribution, and the repeated measures design which allowed each subject to serve as his or her own control in the estimation of the differences in blood pressure due to seasonal effect. The latter aspect is likely to have produced less biased estimates than those obtained from cross-sectional studies, where the seasonal effect is estimated comparing measurements of blood pressure between different individuals, creating room for the confounding effect of interindividual differences.

From the analytical point of view, the use of a random intercept model within the framework of structural equation modeling allowed for a better control of the potential bias due to unrealistic assumptions of homogeneity across individuals; a reduction of the effect of measurement error in blood pressure; and an efficient treatment of missing data under the relatively weak assumption that they are missing at random conditional on the observed covariates. The use of a validated method of adjustment for antihypertensive treatment should also have contributed to bias reduction.

## LIMITATIONS

Among possible confounding factors, this study did not take into account the variability of blood pressure measurement due to its circadian rhythm.^47^ In absence of any specific reason suggesting a relationship between time of day of measurement and season, it is plausible that the omission of this factor produced a bias toward the null of the estimated seasonal effect, thus reinforcing our finding of a strong and statistically significant seasonal effect.

Indoor temperature during measurement has also been shown to affect blood pressure^16^ and was not recorded during the NIDS study. However, in a large-scale population study where blood pressure measurements were taken at the respondents’ homes and not in a controlled environment, it is plausible to assume a causal correlation between indoor temperature and season. In this case, indoor temperature acts more as a (partial) mediator of the observed seasonal effect than a confounder, and statistical adjustment for its effect would be questionable. Adjustment for indoor temperature in the MONICA datasets produced only a marginal change (toward the null, as expected) in the estimated seasonal effects.^13^

The analytical form chosen for the seasonal effect assumes a symmetric peak and trough 6 months apart, and does not allow for other possible seasonal patterns (eg, a sharp increase in autumn and steady decline in spring and summer). However, the plausibility of this shape is well supported by the results of large studies which recovered the seasonal effect nonparametrically.^6,7^ Further, the irregular number of measurements in the different months, and especially the low number in December and January, made it inadvisable to use excessively flexible forms for the seasonal effect.

Finally, while suboptimal response and greater attrition rates were observed in some social strata in the NIDS survey, this phenomenon does not automatically create selection bias, especially in analytical studies and when differences in observed characteristics between respondents and nonrespondents are taken into account through appropriate adjustment of sampling weights.^48^ However, we cannot exclude the possibility that unobserved differences between respondents and nonrespondents might have biased the results of our study in an unpredictable way.

## CONCLUSION

The present study found clear evidence of substantial seasonal variation in blood pressure. In both genders, seasonal variation was slightly larger for systolic than DBP. Seasonal effects in SBP (but not in DBP) was significantly greater among older participants. Seasonal effects were highest among subjects with low education, income, or living in informal areas.

Our results indicate that seasonal variations in blood pressure have concrete implications and should be routinely taken into account both in epidemiological research and in clinical practice. From a public health perspective, our findings suggest that interventions to reduce older subjects’ exposure to low temperatures may contribute to reduction of the winter peak in blood pressure observed in the South African population, and the associated increase in cardiovascular risk.
